# Molecular Basis for Mechanical Properties of ECMs: Proposed Role of Fibrillar Collagen and Proteoglycans in Tissue Biomechanics

**DOI:** 10.3390/biom11071018

**Published:** 2021-07-12

**Authors:** Frederick H. Silver, Nikita Kelkar, Tanmay Deshmukh

**Affiliations:** 1 Department of Pathology and Laboratory Medicine, Robert Wood Johnson Medical School, Rutgers, The State University of New Jersey, Piscataway, NJ 08854, USA; 2 OptoVibronex, LLC., Allentown, PA 18104, USA; nuk1@scarletmail.rutgers.edu (N.K.); tmd24895@gmail.com (T.D.)

**Keywords:** collagen, proteoglycans, elastic modulus, energy storage, energy dissipation, decorin, vibrational optical coherence tomography

## Abstract

Collagen and proteoglycans work in unison in the ECM to bear loads, store elastic energy and then dissipate excess energy to avoid tissue fatigue and premature mechanical failure. While collagen fibers store elastic energy by stretching the flexible regions in the triple helix, they do so by lowering their free energy through a reduction in the entropy and a decrease in charge–charge repulsion. Entropic increases occur when the load is released that drive the reversibility of the process and transmission of excess energy. Energy is dissipated by sliding of collagen fibrils by each other with the aid of decorin molecules that reside on the d and e bands of the native D repeat pattern. Fluid flow from the hydration layer associated with the decorin and collagen fibrils hydraulically dissipates energy during sliding. The deformation is reversed by osmotic forces that cause fluid to reform a hydration shell around the collagen fibrils when the loads are removed. In this paper a model is presented describing the organization of collagen fibers in the skin and cell–collagen mechanical relationships that exist based on non-invasive measurements made using vibrational optical coherence tomography. It is proposed that under external stress, collagen fibers form a tensional network in the plane of the skin. Collagen fiber tension along with forces generated by fibroblasts exerted on collagen fibers lead to an elastic modulus that is almost uniform throughout the plane of the skin. Tensile forces acting on cells and tissues may provide a baseline for stimulation of normal mechanotransduction. We hypothesize that during aging, changes in cellular metabolism, cell–collagen interactions and light and UV light exposure cause down regulation of mechanotransduction and tissue metabolism leading to tissue atrophy.

## 1. Introduction

The ability of mammals to locomote, maintain blood flow, prevent foreign bacteria and viruses from entering the body, control internal hydration and temperature, process nutrients, excrete waste materials and structurally support tissues and organs depends on the structure and mechanical properties of the extracellular matrices (ECMs) that support these functions [1,2,3]. Most of the ability of ECMs to withstand tissue loading is a result of the structural stability of collagen fibers while the ability of tissues to dissipate energy in skin and other tissues is related to the ability of proteoglycans and fluid to reversibly rearrange during loading and unloading cycles [4]. Many of these processes are influenced by mechanotransduction via cell–ECM matrix cross talk. The cross talk involves feedback balancing mechanical forces and chemical signals that are mediated by fibrils, cellular, and cytoskeletal components [4]. An example of this coordination between cellular and macromolecular components working in unison involves energy storage, transmission and dissipation in musculoskeletal tissues [5,6,7,8].

The muscle–tendon unit is an important mechanical element that supports locomotion in mammals [5,6,7,8] by storing elastic energy in the leg tendons and feet of animals [5,6]. Elastic energy is stored during tendon stretching as a result of the force generated by muscle fibers during normal gait [6,7]. Elastic recoil, primarily by collagen fibers in tendons, converts most of the stored energy back to kinetic energy that is used to create joint movement [6,7]. Energy storage capabilities of tendons as well as the ability to measure these properties non-invasively in living humans gives us a wealth of information about how ECMs function under normal physiologic conditions. 

While energy storage is an important component of tissue function, energy dissipation is also important for tissues such as skin to prevent premature mechanical failure through tearing [8]. Energy dissipation involves the interfibrillar matrix found between collagen fibers and is almost completely lost during decellularization of human dermis [9,10]. Information on the molecular basis of energy storage and dissipation is needed to understand why tissue properties change during aging and in disease states.

**Figure 1:**
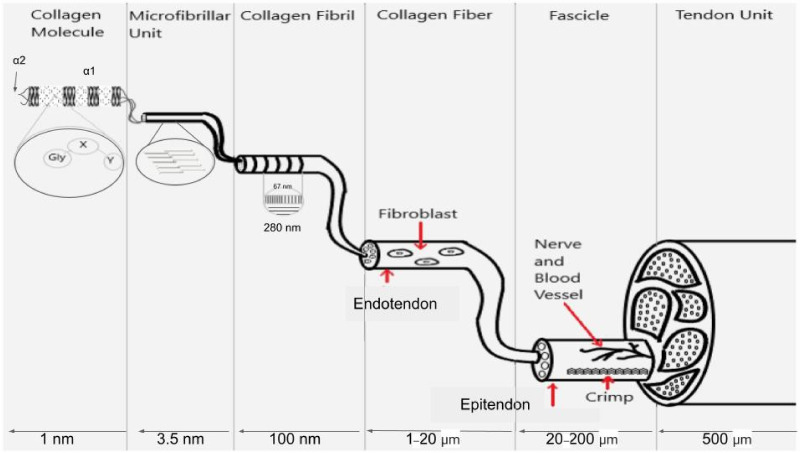
Illustration showing the molecular, microfibrillar, fibrillar and fascicular structure of tendon. Collagen molecules are composed of flexible Gly-X-Y (light regions on left of collagen molecule) sequences that alternate with rigid sequences of Gly-Pro-Hyp (dark regions of collagen molecule) that make a triple helix that is a hybrid of flexible and rigid mechanical elements. The microfibril is composed of 5 quarter-staggered collagen molecules with all the flexible and rigid regions in register. The 67 nm repeat period (D period in tendon) occurs at the fibrillar level when observed in the electron microscope and has all the flexible and rigid regions laterally aligned. Fibroblasts form longitudinal columns of cells on the surface of collagen fibrils. Collagen fibers are made up of laterally fused collagen fibrils and are wrapped with an ECM termed endotendon. Collagen fibers form fascicles and are bound into tendon units by additional ECM termed epitendon. The flexibility of collagen molecules is preserved in the D period of collagen fibrils and fibers and provides the energy storage to the ECM.

The energy storage properties of tendons are a direct consequence of the molecular structure and properties of collagen molecules and fibrils that are connected to muscle fibers. Collagen type I is a triple helical molecule that forms microfibrils, fibrils, fibers and fascicles in tendons (see Figure 1). Fibril forming collagens self-assemble into cross striated units with a characteristic 64–67 nm repeat period seen by electron microscopy [11]. All fibril forming collagens share a triple helical region that is roughly 1000 amino acid residues long with a length of about 300 nm composed of alternating flexible and rigid regions [12].

The semi-flexible rod-like behavior of type I collagen was first established based on measurement of physical properties [13,14] and electron microscopy [15,16,17]. Electron micrographs of the collagen molecule exhibit numerous bends [16] which give rise to its flexibility. Results of modeling studies suggest that sequences without rigid amino acid residues (proline and hydroxyproline) are able to form internal loops [16,17] that give these regions more flexibility. Five flexible sites were identified by Hofmann et al. [17] based on electron microscopic images; they occur at characteristic distances of 30–45, 90–105, 150–157.5, 210–217.5 and 270–277.5 nm from the C terminus of the molecule. Free energy analysis and consideration of electrostatic and entropic considerations suggests that reversible stretching of these flexible regions is consistent with the tensile stress–strain properties of collagen fibers [18].

X-ray diffraction studies on tendon indicate that up to a strain of 2%, molecular stretching along the triple helical axis is the predominant mechanism for deformation [19]. Beyond 2%, axial molecular stretching and intermolecular slippage occur, resulting in increases in both the D period (see Figure 1) and the axial rise per amino acid residue along the triple helix [19,20]. Reversible intermolecular slippage occurs as a result of stretching of the end-to-end molecular crosslinks in collagen fibrils in the tendon. The slope of the elastic stress–strain curve for rat tail tendons corrected for the ratio of the axial rise per residue divided by the macroscopic strain is consistent with the elastic stiffness or spring constant for collagen type I [18,21]. The slope of the viscous stress–strain curve reflects interfibrillar shear interactions and can be used to calculate an “effective” fibril length [21]. Crosslinking increases the elastic spring constant of collagen and the effective fibril length, although it may also limit the increase in the collagen D period as the macroscopic strain increases [21,22]. The shear interactions between collagen fibrils are influenced by the presence of decorin, a leucine rich proteoglycan (PG) that has been shown to increase the tensile strength of collagen fibers in vitro [22,23]. The presence of PGs that bind a hydration layer to the surface of collagen fibrils appear to be important in energy dissipation during tensile loading.

There are three PGs that have been implicated in the mechanical properties of ECMs; they are decorin, biglycan and aggrecan. Decorin, a dermatan sulfate containing PG, binds to the D period of collagen fibrils [24] and links neighboring fibrils in parallel register [25]. Biglycan is another extracellular dermatan sulfate containing PG that has proangiogenesis and proinflammatory properties [24]. Aggrecan, is a highly charged PG that forms aggregates together with hyaluronan and link protein that resist compressive deformation of cartilage [24]. These large aggregates generate a charged densely packed, hydrated network attached to collagen fibrils. PG molecules provide a layer of hydration around collagen fibrils that helps to transfer and dissipate energy during tissue mechanical loading.

Mechanical studies on tendons from mice deficient in decorin and biglycan indicate a reduction in tensile strength and moduli, and increased relaxation times and tissue viscosity [25,26]. In the absence of biglycan, mouse tendon healing is impaired while in the absence of decorin healing is diminished [26]. 

Decorin deficient cartilage exhibits a substantial reduction in aggrecan content that results in a decreased modulus, elevated hydraulic permeability, and reduced energy dissipation capabilities [27]. Aggrecan, due its high surface charge increases cartilage osmotic pressure and can contribute 30–50% to the apparent compressive stiffness of hyaline cartilage [28]. Loss in PG staining is associated with a major reduction in energy storage and dissipating ability of human osteoarthritic cartilage [29,30]. Further studies are needed to better understand the mechanical changes seen in ECMs associated with aging and connective tissue diseases. 

In this paper a model is presented on the molecular structure of collagen and PGs that leads to their ability to store and dissipate energy. In addition, a new method is presented, termed vibrational optical coherence tomography (VOCT), to non-invasively measure elastic energy storage and dissipation in vivo. Based on the results reported in this paper we hypothesize that the modulus of skin is dependent both on the direction of the collagen fibers in the dermis as well as the cell-collagen interactions that are present that oppose the stress along the collagen fibers.

## 2. Methods

### Subjects

In vivo measurements on skin from the arms and legs of 25 subjects (25 to 71 years old) were used as controls for skin stretched 5% from 3 subjects (2 males, 24 and 71 years old and 1 female, 25 years old) after informed consent was obtained. Tissues examined after stretching 5% include skin from the arms and legs and measurements were made at different angles to the lines of maximum tension defined as the angle where the maximum stiffness was measured. Skin was stretched by applying tape to the skin surface and measuring the change in dimensions.

Measurement of Resonant Frequency and the Elastic Energy Storage Ability of ECM Components

Vibrational optical coherence tomography (VOCT) is a non-invasive and non-destructive method that uses audible sound to create a transverse displacement of a tissue as described previously [31,32,33,34]. The displacement of the tissue is detected by measuring the frequency dependence of the deformation based on the reflected infrared light. The result is a spectrum of displacements as a function of frequency of the applied sound. The resonant frequency of a tissue component is defined as the frequency at which the maximum displacement is observed. The measured resonant frequencies are converted into elastic moduli using a calibration equation (Equation (1)) developed based on in vitro uniaxial mechanical tensile testing and VOCT measurements made on the same material [31,32,33,34]. The elastic modulus along with the tissue displacement is a measure of the energy storage capability of a tissue.

Soft Tissues→E × d= 0.0651 × (fn^2^) + 233.2 (1)

The resonant frequency of each sample is determined by measuring the displacement of the tissue resulting from sinusoidal driving frequencies ranging from 30 Hz to 500 Hz, in steps of 10 Hz. The peak frequency (the resonant frequency), fn, is defined as the frequency at which the displacement is maximized as discussed previously [31,32,33,34]. The resonant frequencies and moduli of skin components previously measured using VOCT are listed in Table 1 [31,32,33,34]. Statistical analysis of the moduli of unstretched and stretched skin was conducted using a 2 tailed Student t test. A *p*-value of less than 0.05 was considered a significant difference. All experimental data are listed in the Supplementary Data Tables.

Calibration studies using in vitro uniaxial tensile testing and VOCT measurements were used to develop Equation (1) for soft tissues. Since most soft tissues have a density very close to 1.0, Equation (1) is valid for the majority of tissues found in the body; where the thickness d is in m and is determined from OCT images: fn^2^ is the square of the resonant frequency, and E is the elastic modulus in MPa. Energy storage is obtained from the area under the modulus versus strain curve by measuring the modulus at different strains.

Measurement of Loss Modulus as a Fraction of the Elastic Modulus and Energy Dissipation by ECMs

Viscous component measurements are reported as a percentage (%) of the elastic modulus in MPa. Samples are subjected to three pulses of audible sound sine waves at frequencies between 30 and 500 Hz in steps of 20 Hz. The viscous component of the viscoelastic behavior is obtained from the driving frequency peak by dividing the change in frequency at the half height of the peak (i.e., 3 db down from maximum peak in the power spectrum) by the driving frequency. This method is known as the half-height bandwidth method discussed previously [10]. The energy dissipated by skin is obtained from the area under the viscous modulus (as a % of the elastic modulus) versus strain curve.

## 3. Results

**Figure 2:**
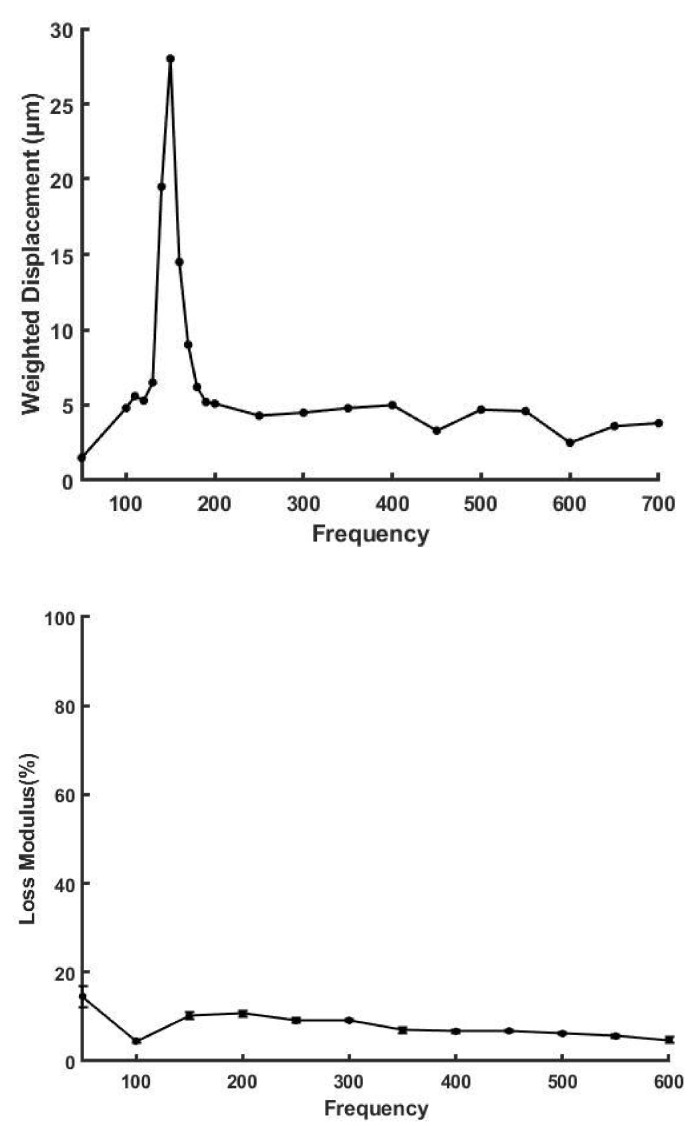
Weighted displacement in μm versus frequency in Hz (**top**) and loss modulus as a percent (%) of the elastic modulus versus frequency for decellularized human skin determined in vitro using VOCT (**bottom**). Decellularized human skin is composed of primarily type I collagen fibers. Note the loss modulus of decellularized skin is very low in comparison to human skin due to the removal of proteoglycans and cells during processing.

VOCT was used to measure the elastic moduli and viscous losses of skin at different angles to the lines of tension in the skin. To plastic surgeons these lines are generally referred to as Langer’s lines. Skin tension is a reflection of the tension in the collagen fibers as well as the tension exhibited by cell-to-collagen adhesion. Figure 2 shows a plot of weighted displacement versus frequency (top) and % loss versus frequency for decellularized human skin measured in vitro (bottom). Decellularized skin is composed predominantly of type I collagen after all the cells and proteoglycans are removed [9,34,35]. The presence of a single resonant frequency peak supports the conclusion that the material is composed of a single element with a narrow molecular weight distribution [9].

**Figure 3:**
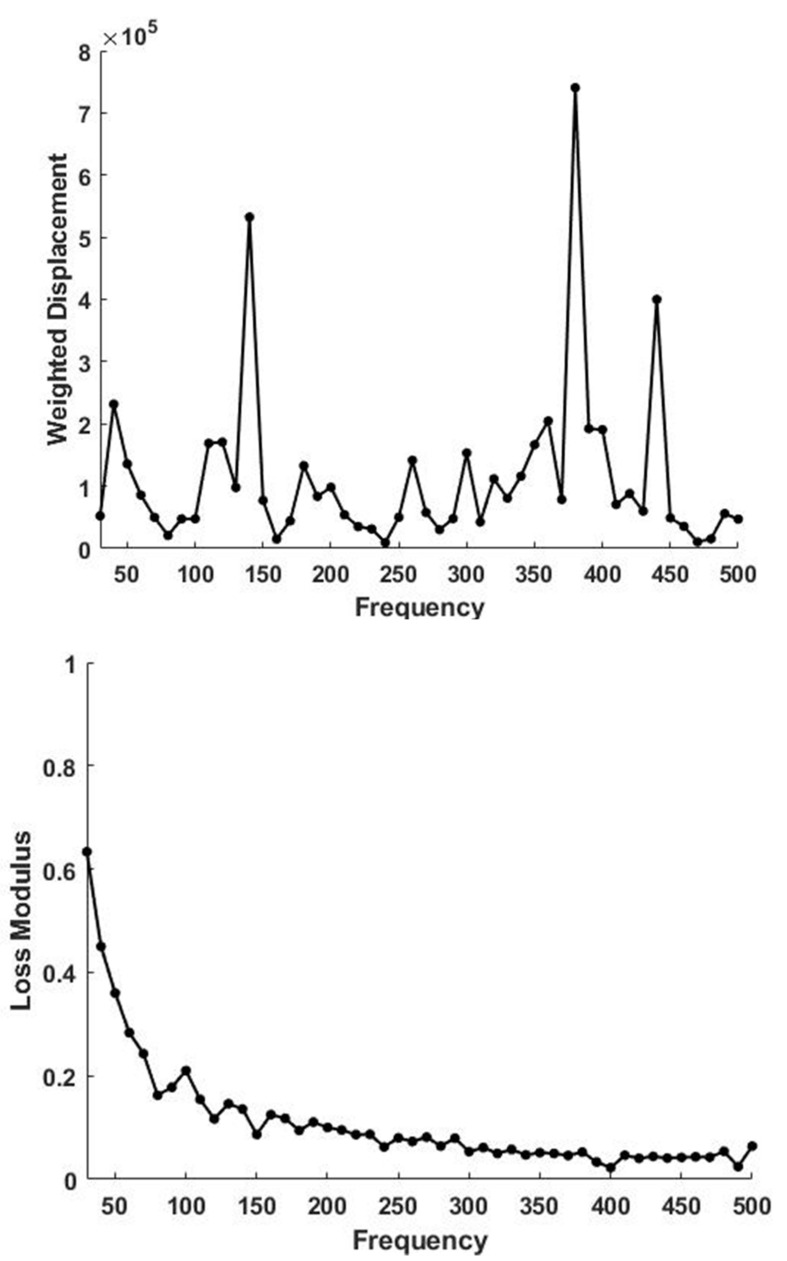
Weighted displacement in μm vs. frequency in Hz for skin determined in vivo using VOCT (**top**). Note the presence of a normal dermal collagen peak at 120 Hz. The peaks at 70, 140, 380 and 440 Hz are due to the cellular contribution, vascular, muscle and tendon contributions based on Table 1. A plot of loss modulus as a % of the elastic modulus versus frequency measured for skin over the biceps muscle is shown (**bottom**). Note the loss modulus is maximized at low frequencies and is much higher than that of decellularized skin.

In comparison, the weighted displacement versus frequency peaks for human skin (Figure 3 top) contains peaks at 50 to 70 Hz, 100 Hz and 150 Hz representing cells, dermal collagen and blood vessels as previously reported [33]. At low frequency the % loss versus frequency plot shown in Figure 3 bottom shows a loss of about 65% at 30 Hz compared with about 12% for decellularized skin. The resonant frequency and stiffness of decellularized skin, skin and other tissue components are listed in Table 1. Removal of cells and PGs results in a loss in energy dissipating ability as indicated by the decreased viscous behavior of decellularized skin in vitro compared to skin in vivo.

**Figure 4:**
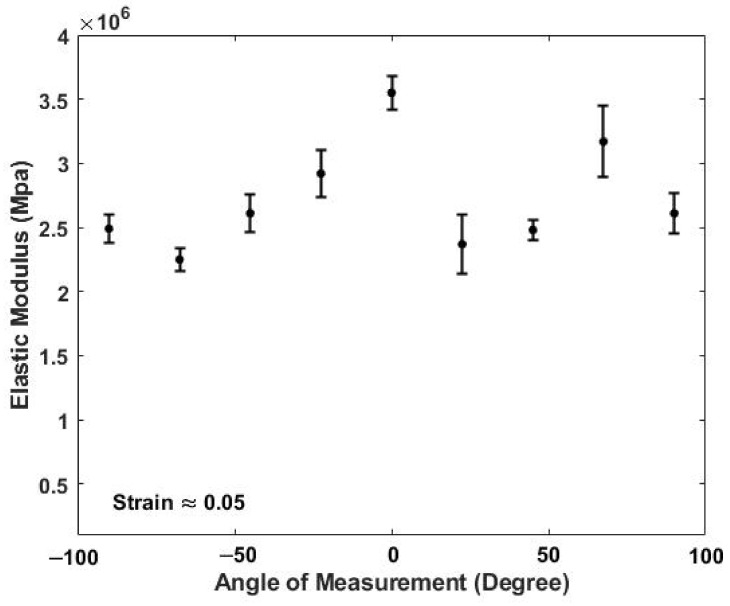
Elastic modulus in MPa of skin versus angle in degrees measured at a strain of 5% in vivo using VOCT. The angle at zero degrees is defined arbitrarily as the angle where the maximum elastic modulus occurs. The moduli measured at 0° and 67.5° are significantly higher than the moduli at all other angles at 5% strain (see Table 2). While under physiologic conditions the collagen fibers in skin are more randomly oriented within the plane of the skin, under external loads the fibers appear to orient at 0° and 67.5°.

Figure 4 shows a typical plot of the in vivo modulus of skin versus angle of measurement at a strain of 5%. The 5% strain is in addition to the strain already present in the tissue which has been estimated to be about 5% [33]. The zero-degree angles are arbitrarily defined as the angle where the modulus is maximized at a strain of 5%. The highest moduli are observed after stretching when the direction of tension is nearest to the orientation of the stretched collagen fiber axes. The statistical analysis of this data shows that the moduli of control unstretched skin and skin stretched 5% at all angles except at 0° and 67.5° are not significantly different (Table 2 and Supplemental Data in Table S1). Stretching assists in defining the collagen fiber angle at 0° and 67.5° since orientation helps align the fibers with the tensile direction. When no external strain is applied to the skin, the modulus does not change with angle of measurement and is about 2.34 MPa suggesting that the collagen fiber orientation is more random in the plane of the skin under normal physiological conditions. The absence of a strong directional dependence of the collagen fibers in unstretched skin allows energy dissipation equally in all directions within the plane of the skin.

**Figure 5:**
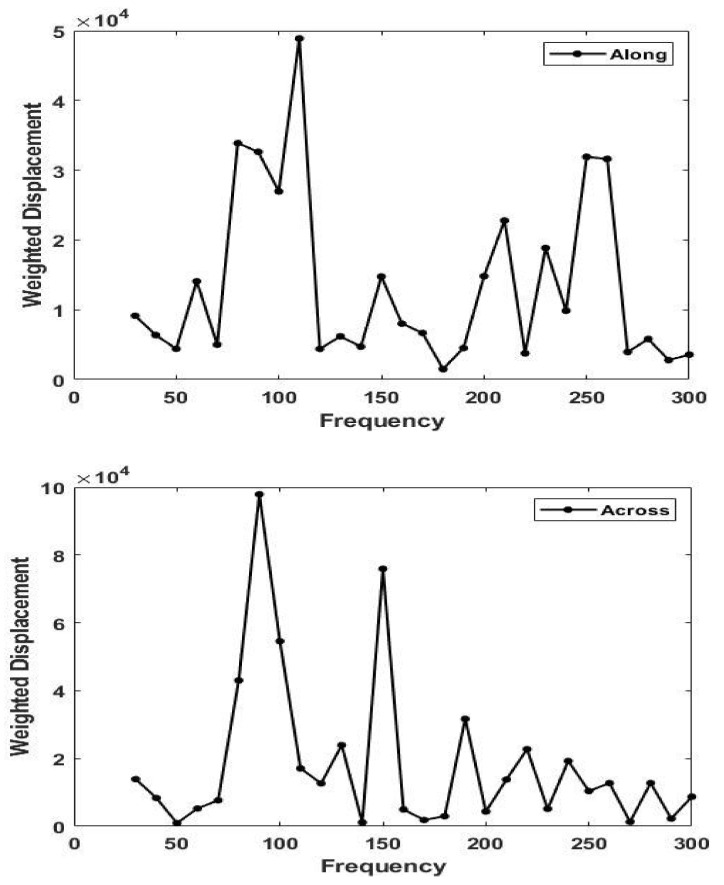
Plots of weighted displacement in μm versus frequency in Hz parallel (0°) to the orientation of the collagen fibers (**top**) and perpendicular (90°) to the orientation of the collagen fibers (**bottom**) in skin in vivo at 5% strain. Note the shift in the dermal collagen peak from about 90 Hz (perpendicular) to 110 Hz (parallel). The modulus measured parallel to the collagen fibers is higher than that perpendicular to the fibers (see Equation (1)).

Figure 5 shows a plot of weighted displacement versus frequency for skin along (0°) and perpendicular (90°) to the direction of maximum tension. Note the difference in the resonant frequency for skin stretched along and perpendicular to the line of maximum tension at 5% strain. The modulus is maximized along the lines of tension and varies from about 2.34 MPa at zero strain to about 3.5 MPa at 5% strain.

**Figure 6:**
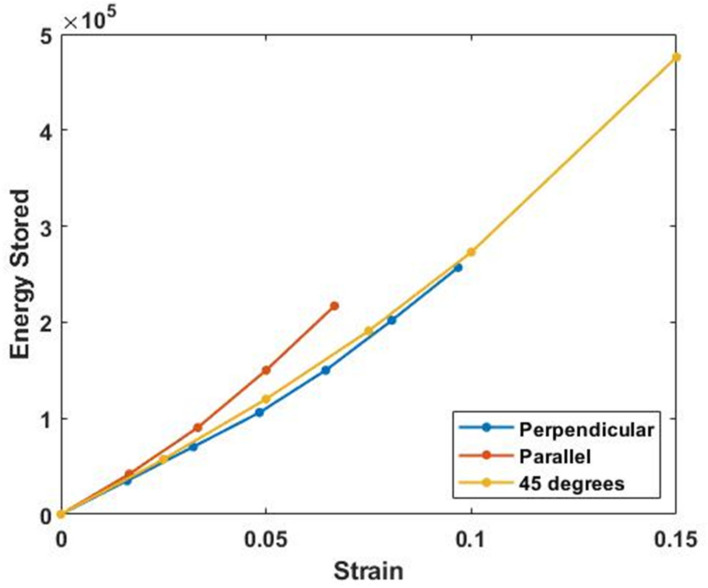
Energy stored parallel (0°), at 45°, and perpendicular (90°) to the collagen fiber orientation in vivo calculated from the area under the modulus-strain curves (with units of MPa). The external strain was applied by stretching the skin with a piece of tape. Note the energy stored in skin appears almost independent of the angle of orientation.

**Figure 7:**
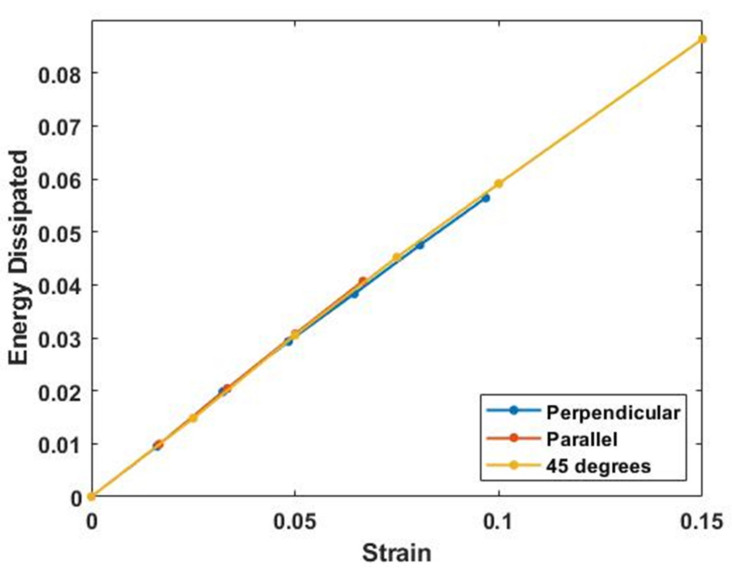
Energy dissipated by skin calculated from the area of the % of elastic modulus versus strain curve (with units of MPa) in vivo as a function of strain at 30 Hz (the lowest frequency that VOCT measurements can be made) and collagen fiber orientation. Note the energy dissipated appears similar for all collagen orientations and only depends on the degree of stretch. Parallel measurements were made at 0° while perpendicular ones were made at 90°.

Figure 6 and Figure 7 show plots of energy stored and energy dissipated at 30 Hz versus strain at different angles with respect to the collagen fibers. The energy stored is calculated as the area under the elastic modulus–strain curve while the energy dissipated is calculated from the area under the loss modulus–strain curve. Note the energy stored and dissipated is almost independent of angle for strains up to about 5%. This allows energy storage and dissipation to occur equally in all directions at low strains within the plane of the skin.

## 4. Discussion

While a great deal of research had provided basic information on the biochemistry and biophysics of ECMs in health and disease, there is a need to relate this information to an understanding of how these changes influence in vivo mechanical properties and physiology of tissues. Changes to the structure and mechanical properties of ECMs have been observed during development and aging and are associated with connective tissue diseases [35,36,37]. Changes in structure not only affect the energy storage capabilities of ECMs but they can lead to premature mechanical failure due to limited ability of tissues to dissipate energy effectively. This is the reason that scar tissue tears more easily than normal skin.

Tendon and ACL failures are well known consequences experienced by athletes due to overloading. The lack of effective energy dissipation leads to wearing away of the cartilage that lines joint surfaces as well as development of skin tears in the elderly. Additional research is needed to understand the consequences of changes in mechanical properties that occur in vivo. The first problem involves the ability to non-invasively measure modulus values of tissue components in vivo while the second is to be able to understand the significance of the normal angular dependence of the modulus along and perpendicular to the axis of stretching of skin and how this relates to the biochemistry of the components that make up each tissue.

### 4.1. Langer’s Lines and the Direction of Collagen Fibers in Skin

In the 1860s Langer first recognized that there were lines of tension in collagen fibers in the skin which cause a circular hole to become elliptical in cadaver skin. These lines have been referred to as Langer’s lines and give the rough orientation of the collagen fibers in the skin at a particular location when the cells are not providing tension [4,38]. Collagen fiber orientation is reported to occur at angles of about ±10° to Langer’s lines [38]; however, in live skin our results suggest that the collagen fibers are more randomly oriented in the plane of the skin due to cellular residual tension [38]. The tension in skin is due both to the forces present in collagen fibers and the retractile forces generated by fibroblasts when they are stretched. For this reason, circular biopsies remain circular and do not become elliptical in skin from living subjects.

The results presented in this study suggest that the tension in living skin is a bit more complicated than Langer considered [4,38]. Langer did not consider the cellular tension and therefore Langer’s lines may not accurately portray the mechanical loading under normal physiological conditions. This may explain why Langer’s lines of tension vary in diagrams presented by different authors and from different anatomic locations. 

**Figure 8:**
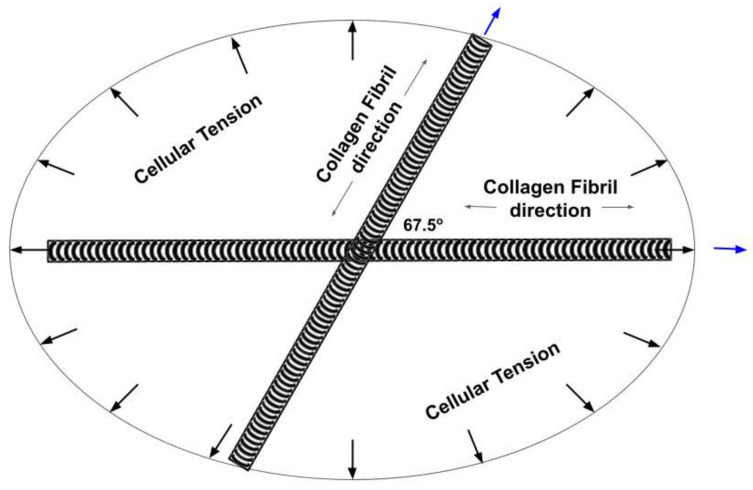
Diagram of the collagen fiber orientation determined from VOCT measurements made on living skin under an applied strain of 5%. When stretched, the collagen fibers attempt to align with the load direction while the cellular tension provides a force to balance the axial fiber forces. When the external load is removed, the cellular tension restores the skin shape by providing a retractile force. Under normal physiologic conditions the collagen fibers are more randomly oriented in the plane of the skin. When the cellular tension is lost, circular defects assume the elliptical orientation determined by the collagen fibers shown in Figure 8.

Figure 8 is a model of the orientation of collagen fibers in the plane of the skin after stretching 5% based on the experimental results presented in this paper. Note the stretched collagen fibers are oriented at 0° and 67.5° from the axis of maximum modulus at 5% strain. When the cellular tension is released the collagen fibers orient along the axes of the collagen fibers (Figure 8) which forms an ellipse. Under physiological conditions the collagen fibers are more randomly oriented within the plane of the skin due to the cellular tension that provides equal resistance to stretching in all directions. 

Since the results reported in this paper suggest that the modulus of skin is almost independent of direction under normal physiological conditions, except when the skin is stretched (see Figure 8), this suggests that cells under an external mechanical load provide a retractive tensile stress that operates uniformly within the plane of the skin. 

**Figure 9:**
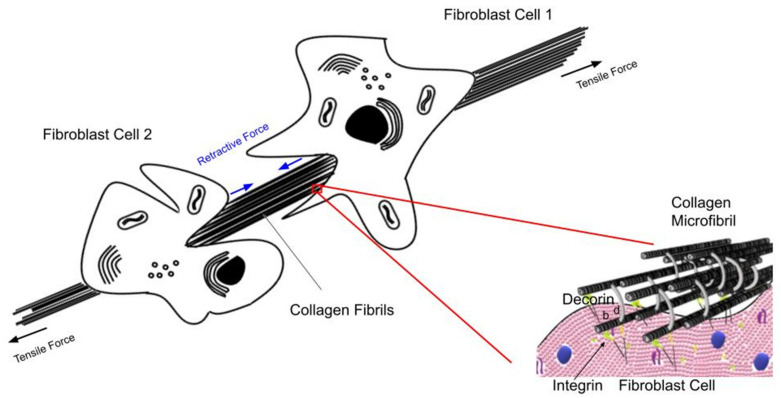
Diagram illustrating the tensile forces acting on collagen fibers and cells acting in the skin. Collagen fibers are stretched in tension under normal physiological conditions. This causes the fibroblasts attached to the collagen fibers to generate a retractive force via attachment through integrin and decorin molecules. These attachments serve as a feed-back mechanism to skin cells reflecting the current level of loading. Increased external loading stretches the collagen fibers as well as the attached cells up-regulating mechanotransduction under normal physiological conditions. This explains why weightlifting increases muscle size and also generates more skin to cover the larger muscles due to fibroblast loading via increases in skin tension. Note the role of integrin–collagen–fibroblast attachments as well as collagen–decorin attachments at the d and e bands.

Figure 9 is a diagram showing the tension in the collagen fibers in the skin under normal physiological conditions as well as the retractive force exerted by fibroblasts sitting on the surface of the collagen fibers. We hypothesize that under normal physiological conditions the tension in the collagen fibers provides a resting point for maintaining normal mechanotransduction and normal tissue turnover. Moderate increases in external loads up-regulates protein synthesis and cellular division. Extreme loading, cellular transformation or trauma leads to tissue failure: while decreased loading, exposure to UV light and aging results in tissue atrophy. 

In this manner fibroblasts attached to the surface of collagen fibers (see Figure 9) prevent defects in skin that are circular from becoming elliptical. In addition, decreases in collagen and cellular tension in skin wounds could putatively lead to upregulation of mechanotransduction and result in anabolic responses including cellular division and protein synthesis that are key to the repair process.

### 4.2. Determination of the Orientation of the Collagen Fibers in Skin

The results of our studies indicate that the collagen fibers in skin appear to orient with the loading direction thus maximizing the ability of skin to store and dissipate energy at any angle in the plane of the skin. The angle between the fibers is about 67.5° degrees at a 5 % strain due to orientation that occurs with stretching (Figure 8). This orientation results in the modulus being almost independent of the direction of loading and suggests that the modulus of the collagen fibers in resting skin is about 2.34 MPa. In addition, a cellular induced tension of up to about 1.0 MPa must occur when the fibers are stretched along their axis. Thus, the cellular tension in skin is superimposed on the collagen tension in skin when living skin is strained. This increases the stress in the collagen fiber network and causes the fibroblasts to try to actively contract the collagen fibers that they are attached to.

### 4.3. Energy Storage and Dissipation by Skin

**Figure 10:**
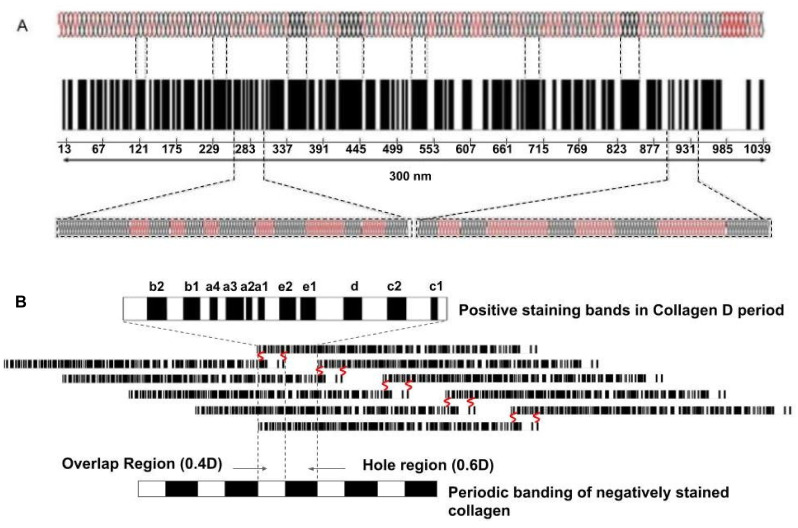
A model depicting the collagen molecule composed of rigid (black) and flexible (red) regions (**A**) and a quarter-staggered array of 5 molecules packed laterally that make up the microfibril (**B**) in the tendon. The numbers under the molecular bar code illustrated in A are the amino acid residue numbers. The flexible and rigid regions shown in A are blown up below the bar code. Initially, under stress the red regions of the molecule are stretched. The banding pattern of the collagen D period is shown in B. The bands a through e represent the positively staining bands characteristic of collagen fibrils seen by electron microscopy that arise from alignment of all the flexible regions in the collagen molecules. The negatively staining bands, showing the hole and overlap regions at the bottom of B, illustrate the penetration of stain into the region of the microfibril devoid of amino acids.

Figure 10 illustrates models of the mechanisms of energy storage and dissipation that occur during stretching of collagen molecules and microfibrils. At low applied strains, 2% or less, the flexible regions in the collagen triple helix are stretched. At higher strains, the triple helix, crosslinks, and D period increase in length.

The area under the modulus–strain curve is related to energy storage of a tissue: while the area under the loss modulus–strain curve is related to the energy dissipation. This area is independent of strain and the direction of testing at low strains as is indicted by our experimental results. This allows skin to stretch in any direction within the plane of the skin without tearing. The ductility of skin in vivo is much less than the ductility of skin measured in vitro due to the presence of cellular stresses that act in all directions within the plane of the living skin. This allows energy to be stored at low strains in all directions of normal skin without tearing occurring. In contrast, scar tissue tears more easily than normal skin because of the uniaxial alignment of the collagen fibers and the more random arrangement of the fibroblasts in scar tissue [39].

### 4.4. Effects of Aging

Histologic changes in skin associated with aging include atrophy of the epidermis, flattening of the dermal–epidermal junction, and thinning of the dermis [40,41,42]. In photoaged skin increased epidermal thickness and severe dermal connective tissue damage occur including accumulation of elastin-containing material, known as solar elastosis [40,41,42,43,44,45].

As the dermis thins with age, new collagen production declines, as well as production of hyaluronic acid and PGs containing dermatan sulfate such as decorin and biglycan [43]. Thus, the undulations, termed rete pegs, that occur at the epidermis–dermis interface, disappear and the associated cutaneous blood flow decreases [40,41,42,43]. Dermal fibroblasts express increased levels of collagen-degrading matrix metalloproteinases-1 (MMP-1) in aged (>80 years old) compared with young (21 to 30 years old) human skin in vivo that leads to fragmentation of collagen fibrils [40,41,42,43]. Young skin has abundant, tightly packed, and well-organized intact collagen fibrils; collagen fibrils in aged skin are fragmented and coarsely distributed [44,45]. Reactive oxygen species generated in the aging process increase metalloproteinase expression and inhibit TGF-β signaling, which leads to collagen fragmentation and decreased collagen biosynthesis. This hinders the mechanical interaction between fibroblasts and the ECM, and consequently leads to a reduction in the size of dermal fibroblasts [46]. Alteration of fibroblast shape regulates c-Jun/AP-1-dependent expression of MMP-1 (metalloproteinases) and consequent collagen fibril fragmentation [47]. Thus, changes in collagen fiber integrity as well as alteration of fibroblast metabolism and fibroblast-collagen interactions may cause aging effects that have mechanical consequences.

Our results suggest that the cellular and collagen tension in skin provides an important mechanism for cell-collagen maintenance of tissue metabolism. In this manner tension both internal to the collagen fibers and external to skin can maintain levels of homeostasis through normal ECM turnover via mechanotransduction. Loss of tension due to tearing or formation of cuts up-regulates tissue formation while ageing and sun damage leads to down–regulation of mechanotransduction and tissue atrophy.

### 4.5. Energy Storage and Dissipation in Tendon

In tendon, the changes with age are not similar to all the changes seen in skin [46]. In some animals the cell density is reduced [48]; however, in horse tendon there is reported to be no difference in cell metabolism and collagen synthesis [49]. Down regulation of matrix remodeling and a reduction of blood vessels are reported in some animals [48]. Associated with tendon injury is rounding of fibroblasts, increases in cell numbers, proteoglycans, water, vascularization and deposition of disorganized collagen fibrils [48]. 

Loading induces a tensile stretch to tenocytes, activating protein kinases [49] and various biologic responses. Physiological exercise has been shown to increase turnover of type I collagen and promote an anabolic response [49]. In contrast, overloading or underloading has been shown to have detrimental effects on the tendon, resulting in a biologic response that is catabolic [49,50,51,52,53]. Studies have shown that loading history affects the mechanical sensitivity of tenocytes, causing a change in their response to the same applied strain [52,53,54] presenting further complexity to the relationship between tissue load and biologic response. The method by which mechanical modulations from the ECM translate into biochemical signals that drive the biological response of the tendon are not well understood; however, the relationship between collagen fiber stresses and fibroblast loading may be very important in understanding mechanotransduction and aging in tendon.

## 5. Conclusions

Collagen and proteoglycans work in unison in the ECM to bear loads, store elastic energy and then dissipate excess energy to avoid tissue fatigue and premature mechanical failure. While collagen fibers store elastic energy by stretching the flexible regions in the triple helix, they do so by lowering their free energy through a reduction in the entropy and a decrease in charge-charge repulsion. Entropy increases occur when the load is released reversing the process and making possible the transmission of excess energy to other tissue components. Energy is dissipated by sliding of collagen fibrils by each other with the aid of decorin molecules that reside on the d and e bands of the native D period. Fluid flow from the layer of hydration associated with decorin and collagen fibrils hydraulically dissipates energy. The deformation is reversed by osmotic forces that cause fluid to reform a hydration shell around the collagen fibrils when the loads are removed.

Collagen fibers in stretched skin are oriented at 0° and 67.5° and are loaded in tension during development and maturation. In addition, tensile retractive forces developed by fibroblasts residing on collagen fibers in dermis balance the tensile forces acting on the collagen fibers. Tensile forces acting on cells and tissues provide a baseline for stimulation of normal mechanotransduction. During aging, changes in cellular metabolism, cell–cell communication, cell–collagen attachments and UV light exposure appear to lead to down regulation of mechanotransduction and tissue metabolism possibly leading to tissue atrophy and enzymatic degradation. 

## Figures and Tables

**Table 1:** Assignment of Resonant Frequency Peaks and Associated Moduli Based on VOCT Measurements [33].

Tissue	Resonant Frequency (Hz) {SD}	Modulus E (MPa) {SD}
Bone		
Lamellar Bone	990 {10.00}	173 {20}
Subchondral Bone	586 {26.07}	67.81 {11.11}
Ear and Lower Nasal cartilage	290 {14.14}	16.2 {1.74}
Upper Nasal Cartilage	380 {14.14}	30.4 {5.89}
Fat, Epidermal Cells	40–70 {12.90}	1.110 {0.25}
Fibrotic Tissue	210 {10}	10.84 {2.48}
Ligament		
Anterior Cruciate Ligament (ACL)	525 {7.07}	53.9 {2.25}
Meniscus	430 {14.14}	31.4 {3.37}
Muscles		
Bicep Muscle	378 {16.02}	29.6 {2.62}
Quadriceps Muscle	365 {21.21}	20.5 {2.32}
Nerve		
Nerve	266 {11.54}	15.86 {2.24}
Normal Skin	110 {7.38}	2.15 {0.29}
Ocular		
Cornea, Sclera	140 {14.14}	2.4 {0.14}
Tendon		
Achilles Tendon	440 {10.00}	34.0 {5.98}
Flexor Digitorum Profundus Tendon	370 {14.14}	22.7 {9.42}
Patellar Tendon	430 {5.77}	33.8 {4.62}
Vascular		
Carotid Artery	136 {11.54}	4.64 {0.98}
Radial Artery	155 {11.98}	3.66 {0.65}
Vein	165 {7.07}	4.84 {0.025}

**Table 2:** Statistical analysis using a 2 tailed t-test of the angular dependence of the modulus of skin (Figure 4). The 0° angle defined as the angle where the maximum modulus is measured. Only the moduli at 0° and 67.5° at 5% strain are significantly different than that of the unstretched skin.

Condition	Average Modulus Value	Standard Deviation	Sample Size	*p* Value	Significance
**Control (No strain)**	2.34 × 10^6^	6.91 × 10^5^	17	NA	NA
**Strain Direction: 0 degree** **(5 Percent Strain)**	3.55 × 10^6^	2.56 × 10^5^	5	1.88 × 10^−4^ *	**Significant**
**Strain direction: 67.5 degree** **(5 Percent Strain)**	3.03 × 10^6^	4.07 × 10^5^	6	1.31 × 10^−2^ *	**Significant**
**Strain direction along 22.5, 45, 90, −67.5, −45, −22.5 degrees** **(5 Percent strain)**	2.54 × 10^6^	3.12 × 10^5^	27	1.56 × 10^−1^	Not Significant

* Significantly Different to value at control (No Strain).

## Data Availability

All data generated in this study will be posted at optovibronex.com.

## Supplementary Materials

The following are available online at https://www.mdpi.com/article/10.3390/biom11071018/s1, Table S1: Supplementary Skin Modulus Measurement Data.

## Abbreviations

ECM	extracellular matrix
PG	Proteoglycan
VOCT	vibrational optical coherence tomography
ACL	anterior cruciate ligament

## References

[1] Silver F.H. (2006). Mechanosensing and Mechanochemical Transduction in Extracellular Matrix.

[2] Franchi M., Trire A., Quaranta M., Orsini E., Ottani V. (2007). Collagen structure of tendon relates to function. Sci. World J..

[3] Holmes D.F., Lu Y., Straborg T., Kadler K.E. (2018). Collagen fibril assembly and function. Curr. Top. Dev. Biol..

[4] Silver F.H., Siperko L.M. (2003). Mechanosensing and Mechanochemical Transduction. Crit. Rev. Biomed. Eng..

[5] Roberts T.J., Marsh R.L., Weyand P.G., Taylor C.R. (1997). Muscular force in running turkeys: The economy of minimizing work. Science.

[6] Alexander R.M. (1983). Animal Mechanics.

[7] Alexander R.M. (1984). Elastic energy stores in running vertebrates. Acta Zool..

[8] Wilson A.M., McGuigan M.P., Su A., van den Bogert A.J. (2001). Horses damp the spring in their step. Nature.

[9] Dunn M.D., Silver F.H. (1983). Viscoelastic Behavior of Human Connective Tissues: Relative Contribution of Viscous and Elastic Components. Connect. Tissue Res..

[10] Shah R.G., Devore D., Silver F.H. (2018). Biomechanical analysis of decellularized dermis and skin: Initial in vivo observations using OCT and vibrational analysis. J. Biomed. Mat. Res..

[11] Shah R.G., Silver F.H. (2017). Viscoelastic behavior of tissues and implant materials: Estimation of the elastic modulus and viscous contribution using optical coherence tomography and vibrational analysis. J. Biomed. Tech. Res..

[12] Hodge A.J., Petruska J.A., Ramachandran G.N. (1963). Recent studies with the electron microscope on ordered aggregates of the tropocollagen macromolecule. Aspects of Protein Structure.

[13] Silver F.H., Horvath I., Foran D.J. (2002). Mechanical implications of the domain structure of fibril forming collagens: Comparison of the molecular and fibrillar flexibility of α-chains found in types I, II and III collagens. J. Theor. Biol..

[14] Fletcher G.C. (1976). Dynamic light scattering from collagen solutions. I. Translational diffusion coefficient and aggregation effects. Biopolymers.

[15] Silver F.H., Langley K.H., Trelstad R.L. (1979). Type I collagen fibrillogenesis: Initiation via a reversible linear growth step. Biopolymers.

[16] Silver F.H., Birk D.E. (1984). Molecular structure of collagen in solution: Comparison of types I, II, III, and V. Int. J. Biol. Macromol..

[17] Paterlini M.G., Nemethy G., Scheraga H.A. (1995). The energy of formation of internal loops in triple-helical collagen polypeptides. Biopolymers.

[18] Hofmann H., Voss T., Kuhn K., Engle J. (1984). Localization of flexible sites in thread-like molecules from electron micrographs: Comparison of interstitial, basement membrane and intima collagens. J. Mol. Biol..

[19] Freeman J.W., Silver F.H., Woods M.D., Laurencin C.T. (2005). The role of Type I collagen molecular structure in tendon elastic energy storage. Mater. Res. Soc..

[20] Mosler E., Folkhard W., Knorzer E., Nemetschek-Gansler H., Nemetschek T.H., Koch M.H. (1985). Stress-induced molecular arrangement in tendon collagen. J. Mol. Biol..

[21] Sasakai N., Odajima S. (1996). Elongation mechanism of collagen fibrils and force-strain relationships of tendon at each level of structural hierarchy. J. Biomech..

[22] Silver F.H., Christiansen D.L., Snowhill P.B., Chen Y. (2001). Transition from viscous to elastic-based dependency of mechanical properties of self-assembled type I collagen fibers. J. Appl. Polym. Sci..

[23] Pins G.D., Christiansen D.L., Patel R., Silver F.H. (1997). Self-assembly of collagen fibers. Influence of fibrillar alignment and decorin on mechanical properties. Biophys. J..

[24] Silver F.H., Freeman J., Seehra G.P. (2003). Collagen self-assembly and development of matrix mechanical properties. J. Biomech..

[25] Iozzo R.V., Liliana Schaefer L. (2015). Proteoglycan form and function: A comprehensive nomenclature of proteoglycans. Matrix Biol..

[26] Kelsey A., Robinsona M.S., Carrie E., Barnuma C.E., Weiss S.N., Huegela J., Shetyea S.S., Linb L., Saezb D., Adams S.M. (2017). Decorin and biglycan are necessary for maintaining collagen fibril structure, fiber realignment, and mechanical properties of mature tendons. Matrix Biol..

[27] Dunkman A.A., Buckleya M.R., Mienaltowski M.J., Sheila M., Adams S.M., Thomas S.J., Satchella L., Kumar A., Pathmanathan L., Beason D.P. (2014). The tendon injury response is influenced by decorin and biglycan. Ann. Biomed. Eng..

[28] Han B., Li Q., Wang C., Patel P., Adams S.M., Doyran B., Nia H.T., Oftadeh R., Zhou S., Li C.Y. (2019). Decorin regulates the aggrecan network integrity and biomechanical functions of cartilage extracellular matrix. ACS Nano.

[29] Mow V.C., Ateshian G.A., Lai W.M., Gu W.Y. (1998). Effects of fixed charges on the stress-relaxation behavior of hydrated soft tissues in a confined compression problem. Int. J. Solids Struct..

[30] Silver F.H., Bradica G., Tria A. (2002). Elastic energy storage in human articular cartilage: Estimation of the elastic spring constant for type II collagen and changes associated with osteoarthritis. Matrix Biol..

[31] Silver F.H., Horvath I., Kelkar N., Deshmukh T., Shah R. (2020). In vivo biomechanical analysis of human tendon using vibrational optical coherence tomography: Preliminary results. J. Clin. Cases Rep..

[32] Silver F.H., Shah R.G., Silver L.L. (2018). The use of vibrational optical coherence tomography in matching host tissue and implant mechanical properties. Biomater. Med. Appl..

[33] Silver F.H., Shah R.G., Richard M., Benedetto D. (2019). Comparative “virtual biopsies” of normal skin and skin lesions using vibrational optical coherence tomography. Ski. Res. Tech..

[34] Silver F.H., Kelkar N., Desmukh T., Horvath I., Shah R.G. (2020). Mechano-Vibrational Spectroscopy of Tissues and Materials Using Vibrational Optical Coherence Tomography: A New Non-Invasive and Non-Destructive Technique. Recent Prog. Mater..

[35] Silver F.H., De Vore D., Shah R. (2017). Biochemical, biophysical, and mechanical characterization of decellularized dermal implants. Mat. Sci. Appl..

[36] Silver F.H., Kato Y.P., Ohno M., Wasserman A.J. (1992). Analysis of mammalian connective tissue: Relationship between hierarchical structures and mechanical properties. J. Long Term Eff. Med. Implants..

[37] Kalath S., Tsipouras P., Silver F.H. (1986). Non-invasive assessment of aortic mechanical properties. Ann. Biomed. Eng..

[38] Kalath S., Tsipouras P., Silver F.H. (1987). Increased aortic root stiffness associated with osteogenesis imperfecta. Ann. Biomed. Eng..

[39] Silver F.H., Siperko L.M., Seehra G.P. (2002). Mechanobiology of force transduction in dermis. Skin Res. Technol..

[40] Dunn M.G., Silver F.H., Swann D.A. (1985). Mechanical analysis of hypertrophic scar tissue: Structural basis for apparent increased rigidity. J. Invest. Derm..

[41] Fisher G.J., Varani J., Voorhees J.J. (2008). Looking older: Fibroblast collapse and therapeutic implications. Arch. Dermatol..

[42] El-Domyati M., Attia S., Saleh F., Brown D., Birk D.E., Gasparro F., Ahmad H., Uitto J. (2002). Intrinsic aging vs. Photoaging: A comparative histopathological, immunohistochemical, and ultrastructural study of skin. Exp. Dermatol..

[43] Fisher G.J., Wang Z.Q., Datta S.C., Varani J., Kang S., Voorhees J.J. (1997). Pathophysiology of premature skin aging induced by ultraviolet light. N. Engl. J. Med..

[44] Lavker R., Gilchrest B. (1995). Cutaneous aging: Chronologic versus photoaging. Photodamage.

[45] Fore J. (2006). A Review of skin and the effects of aging on skin structure and function. Wound Manag. Prev..

[46] Quan T., Fisher G.J. (2015). Role of age-associated alterations of the dermal extracellular matrix microenvironment in human skin aging: A mini-review. Gerontology.

[47] Quan T., Shao Y., He T., Voorhees J.J., Fisher G.J. (2010). Reduced expression of connective tissue growth factor (ctgf/ccn2) mediates collagen loss in chronologically aged human skin. J. Invest. Dermatol..

[48] Jung-Won Shin J.-W., Kwon S.-W., Choi J.-I., Na J.-I., Huh C.-H., Choi H.-R., Park K.-C. (2019). Molecular mechanisms of dermal aging and antiaging approaches. Int. J. Mol. Sci..

[49] Magnusson S.P., Kjaer M. (2019). The impact of loading, unloading, ageing and injury on the human tendon. J. Physiol..

[50] Nagy I.Z., Von Hahn H.P., Verzar F. (1969). Age-related alterations in the cell nuclei and the DNA content of rat tail tendon. Gerontologia.

[51] Langberg H., Rosendal L., Kjaer M. (2001). Training-induced changes in peritendinous type I collagen turnover determined by microdialysis in humans. J. Physiol..

[52] Gardner K., Arnoczky S.P., Caballero O., Lavagnino M. (2008). The effect of stress-deprivation and cyclic loading on the TIMP/MMP ratio in tendon cells: An in vitro experimental study. Disabil. Rehabil..

[53] Archambault J.M., Hart D.A., Herzog W. (2001). Response of rabbit Achilles tendon to chronic repetitive loading. Connect. Tissue Res..

[54] Arnoczky S.P., Tian T., Lavagnino M., Gradner K., Schuler P., Morse P. (2002). Activation of stress-activated protein kinases (SAPK) in tendon cells following cyclic strain: The effects of strain frequency, strain magnitude, and cytosolic calcium. J. Orthop Res..

