# Supplementary material for: Molecular Basis for Mechanical Properties of ECMs: Proposed Role of Fibrillar Collagen and Proteoglycans in Tissue Biomechanics
Source: Biomolecules. 2021 Jul 12;11(7):1018. doi: 10.3390/biom11071018 (PMC8301845; doi:10.3390/biom11071018)
Supplement: Supplementary file 1 [file biomolecules-11-01018-s001.zip › biomolecules-1177016-SI.pdf]

Table S1. Supplementary Skin Modulus Measurement Data.

Control Modulus Measurements on Skin

Age	Sex	Location	Modulus (MPa)	Angle (Degrees)	Strain
26	F	Skin above Gastrocnemius Muscle	2.47	Control *	0
26	F	Anterior Antebrachial Region	2.07	Control *	0
25	M	Anterior Antebrachial Region	2.13	Control *	0
25	M	Skin above Gastrocnemius Muscle	1.920	Control *	0
70+	M	Anterior Antebrachial Region	2.47	Control *	0
25	M	Skin above Gastrocnemius Muscle	2.98	Control *	0
71	M	Anterior Antebrachial Region	4.05	Control *	0
71	M	Posterior Antebrachial Region	2.84	Control *	0
25	F	Posterior Antebrachial Region	2.60	Control *	0
71	M	Anterior Carpal Region	2.48	Control *	0
60+	F	Anterior Carpal Region	2.09	Control *	0
70+	M	Anterior Carpal Region	1.730	Control *	0
34	M	Anterior Carpal Region	2.19	Control *	0
26	F	Anterior Carpal Region	1.900	Control *	0
71	M	Anterior Carpal Region	1.944	Control *	0
62	M	Anterior Carpal Region	2.16	Control *	0
62	M	Anterior Carpal Region	1.838	Control *	0

*** Measurements were made without fixing the Angle**

Angular Modulus Measurement Studies on Skin

Age	Sex	Location	Modulus (MPa)	Angle (Degrees)	Strain
26	F	Skin above Gastrocnemius Muscle	3.34	−22.5	~5%
26	F	Skin above Gastrocnemius Muscle	2.78	−22.5	~5%

26	F	Skin above Gastrocnemius Muscle	2.63	−22.5	~5%
26	F	Skin above Gastrocnemius Muscle	2.89	22.5	~5%
26	F	Skin above Gastrocnemius Muscle	2.21	22.5	~5%
26	F	Skin above Gastrocnemius Muscle	2.03	22.5	~5%
26	F	Skin above Gastrocnemius Muscle	2.55	45	~5%
26	F	Skin above Gastrocnemius Muscle	2.58	45	~5%
26	F	Skin above Gastrocnemius Muscle	2.31	45	~5%
26	F	Skin above Gastrocnemius Muscle	2.28	90	~5%
26	F	Skin above Gastrocnemius Muscle	2.73	90	~5%
26	F	Skin above Gastrocnemius Muscle	2.46	90	~5%
71	M	Anterior Carpal Region	2.57	90	~5%
25	M	Skin above Gastrocnemius Muscle	3.11	90	~5%
26	F	Skin above Gastrocnemius Muscle	2.49	−45	~5%
26	F	Skin above Gastrocnemius Muscle	3.04	−45	~5%
26	F	Skin above Gastrocnemius Muscle	2.37	−45	~5%
25	M	Anterior Carpal Region	2.53	−45	~5%
26	F	Skin above Gastrocnemius Muscle	2.35	−67.5	~5%
26	F	Skin above Gastrocnemius Muscle	2.04	−67.5	~5%
26	F	Skin above Gastrocnemius Muscle	2.31	−67.5	~5%
26	F	Anterior Carpal Region	2.31	−67.5	~5%
26	F	Anterior Carpal Region	2.70	−67.5	~5%
26	F	Anterior Carpal Region	2.37	−67.5	~5%
26	F	Skin above Gastrocnemius Muscle	3.89	0	~5%
26	F	Skin above Gastrocnemius Muscle	3.47	0	~5%
26	F	Skin above Gastrocnemius Muscle	3.20	0	~5%
25	M	Anterior Carpal Region	3.67	0	~5%
71	M	Anterior Carpal Region	3.50	0	~5%
26	F	Skin above Gastrocnemius Muscle	2.78	67.5	~5%
26	F	Skin above Gastrocnemius Muscle	3.11	67.5	~5%
26	F	Skin above Gastrocnemius Muscle	2.76	67.5	~5%
26	F	Anterior Carpal Region	2.53	67.5	~5%
26	F	Anterior Carpal Region	3.45	67.5	~5%
26	F	Anterior Carpal Region	3.54	67.5	~5%
